# Supplementary material for: Pathway for enhanced recovery after spinal surgery-a systematic review of evidence for use of individual components
Source: BMC Anesthesiol. 2021 Mar 10;21:74. doi: 10.1186/s12871-021-01281-1 (PMC7944908; doi:10.1186/s12871-021-01281-1)
Supplement: Supplementary file 1 — Additional file 1: PRISMA Checklist. [file 12871_2021_1281_MOESM1_ESM.doc]

| **#** | **Checklist item** | | **Reported on page #** |
| --- | --- | --- | --- |
|  | |  | |
| 1 | Identify the report as a systematic review, meta-analysis, or both. | | 1 |
|  | |  | |
| 2 | Provide a structured summary including, as applicable: background; objectives; data sources; study eligibility criteria, participants, and interventions; study appraisal and synthesis methods; results; limitations; conclusions and implications of key findings; systematic review registration number. | | 2 |
|  | |  | |
| 3 | Describe the rationale for the review in the context of what is already known. | | 3 |
| 4 | Provide an explicit statement of questions being addressed with reference to participants, interventions, comparisons, outcomes, and study design (PICOS). | |  |
|  | |  | |
| 5 | Indicate if a review protocol exists, if and where it can be accessed (e.g., Web address), and, if available, provide registration information including registration number. | | 3 |
| 6 | Specify study characteristics (e.g., PICOS, length of follow-up) and report characteristics (e.g., years considered, language, publication status) used as criteria for eligibility, giving rationale. | | 4 |
| 7 | Describe all information sources (e.g., databases with dates of coverage, contact with study authors to identify additional studies) in the search and date last searched. | | 4 |
| 8 | Present full electronic search strategy for at least one database, including any limits used, such that it could be repeated. | | Supplementary material |
| 9 | State the process for selecting studies (i.e., screening, eligibility, included in systematic review, and, if applicable, included in the meta-analysis). | | 4 |
| 10 | Describe method of data extraction from reports (e.g., piloted forms, independently, in duplicate) and any processes for obtaining and confirming data from investigators. | | 4 |
| 11 | List and define all variables for which data were sought (e.g., PICOS, funding sources) and any assumptions and simplifications made. | | 4 |
| 12 | Describe methods used for assessing risk of bias of individual studies (including specification of whether this was done at the study or outcome level), and how this information is to be used in any data synthesis. | | 4-thematic synthesis |
| 13 | State the principal summary measures (e.g., risk ratio, difference in means). | | 4-thematic synthesis |
| 14 | Describe the methods of handling data and combining results of studies, if done, including measures of consistency (e.g., I^2^) for each meta-analysis. | | 4-thematic synthesis |

Page 1 of 2

| **#** | **Checklist item** | | **Reported on page #** |
| --- | --- | --- | --- |
| 15 | Specify any assessment of risk of bias that may affect the cumulative evidence (e.g., publication bias, selective reporting within studies). | | 4 |
| 16 | Describe methods of additional analyses (e.g., sensitivity or subgroup analyses, meta-regression), if done, indicating which were pre-specified. | | n/a |
|  | |  | |
| 17 | Give numbers of studies screened, assessed for eligibility, and included in the review, with reasons for exclusions at each stage, ideally with a flow diagram. | | 7 |
| 18 | For each study, present characteristics for which data were extracted (e.g., study size, PICOS, follow-up period) and provide the citations. | | 7 |
| 19 | Present data on risk of bias of each study and, if available, any outcome level assessment (see item 12). | | Risk of bias presented across outcomes with EP tables |
| 20 | For all outcomes considered (benefits or harms), present, for each study: (a) simple summary data for each intervention group (b) effect estimates and confidence intervals, ideally with a forest plot. | | 9-22  Thematic synthesis was presented across outcomes |
| 21 | Present results of each meta-analysis done, including confidence intervals and measures of consistency. | | 9-22  Meta-analysis was not performed in this review of complex intervention |
| 22 | Present results of any assessment of risk of bias across studies (see Item 15). | | Risk of bias presented across outcomes with EP tables |
| 23 | Give results of additional analyses, if done (e.g., sensitivity or subgroup analyses, meta-regression [see Item 16]). | | n/a |
|  | |  | |
| 24 | Summarize the main findings including the strength of evidence for each main outcome; consider their relevance to key groups (e.g., healthcare providers, users, and policy makers). | | 9-22 |
| 25 | Discuss limitations at study and outcome level (e.g., risk of bias), and at review-level (e.g., incomplete retrieval of identified research, reporting bias). | | 22 |
| 26 | Provide a general interpretation of the results in the context of other evidence, and implications for future research. | | 9-22 |
|  | |  | |
| 27 | Describe sources of funding for the systematic review and other support (e.g., supply of data); role of funders for the systematic review. | | 22 |

*From:*  Moher D, Liberati A, Tetzlaff J, Altman DG, The PRISMA Group (2009). Preferred Reporting Items for Systematic Reviews and Meta-Analyses: The PRISMA Statement. PLoS Med 6(7): e1000097. doi:10.1371/journal.pmed1000097

For more information, visit: **www.prisma-statement.org**.

Page 2 of 2
