# Supplementary material for: Pathway for enhanced recovery after spinal surgery-a systematic review of evidence for use of individual components
Source: BMC Anesthesiol. 2021 Mar 10;21:74. doi: 10.1186/s12871-021-01281-1 (PMC7944908; doi:10.1186/s12871-021-01281-1)
Supplement: Supplementary file 2 — Additional file 2: Medline Literature Search. [file 12871_2021_1281_MOESM2_ESM.docx]

Literature Search items

Database: Ovid Medline (R) 1990- January 2020

| 1. Neurosurgical Procedures.mp |
| --- |
| 2. *cervical vertebrae/su [Surgery] |
| 3. *thoracic vertebrae/su [Surgery] |
| 4.*lumbar vertebrae/su [Surgery] |
| 5. Microdiscectomy.mp. |
| 6. Laminectomy.mp. |
| 7. Spinal Fusion.mp. |
| 8. Cervical decompression.mp. |
| 9. Scoliosis.mp. |
| 10. ( spin$ surg$).tw. |

| 11.1 or 2 or 3or 4 or 5 or 6 or 7 or 8 or 9 or 10 |
| --- |
| 12.Chewing Gum/[Enhanced Recovery] |
| 13.Early Ambulation/ |
| 14.Exercise Therapy/ |
| 15. Heating/ |
| 16. Intraoperative Care/mt |
| 17. Preoperative Care/mt |
| 18. Perioperative Care/mt |
| 19.Postoperative Care/mt |
| 20. Patient Education as Topic/ |
| 21.Surgical Procedures, Minimally Invasive/ |
| 22. (accelerat$ adj2 mobil$).tw. |
|  |
| 23.(crystalloid adj manage$).tw.  24.(enhanced adj recover$).tw.  25.ERAS.tw.  26.(fast adj tract$).tw.  27.(fast adj track$).tw.  28.intraoperative.mp. and (IV adj fluid?).tw.  29.intraoperative.mp. and Infusions, Intravenous/  30.(intraoperative and analgesi$).mp.  31.intraoperative.mp. and (fluid adj manag$).tw.  32.(intraoperative and (pain adj manage$)).mp.  33.(postoperative and (fluid adj therap$)).mp.  34.(postoperative and analgesi$).mp.  35.((postoperative adj care) and enhanced).tw.  36.((postoperative adj care) and early).tw.  37.(post-operative and catheter?).mp.  38.(post-operative and (fluid adj therap$)).mp.  39.post-operative.mp. and (fluid adj manage$).tw.  40.post-operative.mp. and (electrolyte$ adj manag$).tw.  41.(post-operative and analgesi$).mp.  42. 12-42  43. 11 and 42  44.exp Humans/ not animals.mp.  45.43 and 44 |
|  |
