# Supplementary material for: Pathway for enhanced recovery after spinal surgery-a systematic review of evidence for use of individual components
Source: BMC Anesthesiol. 2021 Mar 10;21:74. doi: 10.1186/s12871-021-01281-1 (PMC7944908; doi:10.1186/s12871-021-01281-1)
Supplement: Supplementary file 3 — Additional file 3: Evidence Profile Tables. [file 12871_2021_1281_MOESM3_ESM.docx]

**Evidence Profile Tables**

Evidence Profile tables were generated when a number of studies were identified investigating an intervention for one of the predetermined outcomes. We have generated Evidence Profile tables for the following pathway components:

2.4 Tobacco use,

3.Prehabilitation,

4.1Preoperative nutritional screening,

5.Management of anemia,

6.Peri-operative blood conservation strategies,

12. Standard anaesthetic protocol,

16. Perioperative analgesia including use of intravenous lignocaine and

21.Mobilization.

For the following elements we identified published meta-analysis:

6.Peri-operative blood conservation strategies, use of tranexamic acid,

8. Pre-emptive analgesia,

10.2 Antimicrobial prophylaxis,

11. Local anaesthetic infiltration,

13. Surgical access (open and minimally invasive spinal surgery),

16. Perioperative analgesia including use of NSAIDS, ketamine, gabapentinoids and intrathecal morphine and

17. Thromboprophylaxis.

We incorporated the relevant meta-analysis findings into each pathway synthesis of evidence. We rated the risk of bias in the meta-analysis using the AMSTAR meta-analysis rating tool ([1](#_ENREF_1)).

**2. Risk assessment, preoperative optimization, including lifestyle factor modicification**

**2.4 Question**: Do smokers have a higher risk of non-union/surgical complications after surgery of the spine

**Setting**: Perioperative patient management

| **Certainty assessment** | | | | | | | **№ of patients** | | **Certainty** | **Importance** |
| --- | --- | --- | --- | --- | --- | --- | --- | --- | --- | --- |
| **№ of studies** | **Study design** | **Risk of bias** | **Inconsistency** | **Indirectness** | **Imprecision** | **Other considerations** | **Smokers with outcome** | **Non smokers with outcome** |  |  |
| 3  ([2](#_ENREF_2))  ([3](#_ENREF_3))  ([4](#_ENREF_4)) | observational studies | very serious | serious | serious | very serious | strong association all plausible residual confounding would reduce the demonstrated effect | 96/334 | 80/699 | ⨁◯◯◯ VERY LOW | Important |

**3.Prehabilitation**

**Question**: Prehabilitation compared to standard care for patients undergoing and enhanced recovery pathway in surgery of the spine

**Setting**: Perioperative patient care

| **Certainty assessment** | | | | | | | **№ of patients** | | **Effect** | | **Certainty** | **Importance** |
| --- | --- | --- | --- | --- | --- | --- | --- | --- | --- | --- | --- | --- |
| **№ of studies** | **Study design** | **Risk of bias** | **Inconsistency** | **Indirectness** | **Imprecision** | **Other considerations** | **rehabilitation** | **standard care** | **Relative (95% CI)** | **Absolute (95% CI)** |  |  |
| **Pain related outcomes, including VAS/NRS (heterogenous follow up periods)** | | | | | | | | | | | | |
| 3  ([5](#_ENREF_5))  ([6](#_ENREF_6), [7](#_ENREF_7)) | randomised trials | serious | not serious | not serious | serious | all plausible residual confounding would suggest spurious effect; | 190 | 188 | - | - | ⨁⨁⨁◯ MODERATE | Important |
|  | | | | | | | | | | | | |
| **Patient reported outcomes (PROM's)** | | | | | | | | | | | | |
| 4  ([8](#_ENREF_8))  ([6](#_ENREF_6), [7](#_ENREF_7))  ([9](#_ENREF_9)) | randomised trials | serious | not serious | not serious | serious | all plausible residual confounding would suggest spurious effect; | 218 | 196 | - | - | ⨁⨁⨁◯ MODERATE | Important |

**4. Pre-operative nutritional care**

**4.1 Question**: Nutritional screening compared to no screening in patients undergoing spinal surgery

**Setting**: Peri-operative care setting

| **Certainty assessment** | | | | | | | | | | | | | | | **№ of patients** | | | **Certainty** | | **Importance** | |
| --- | --- | --- | --- | --- | --- | --- | --- | --- | --- | --- | --- | --- | --- | --- | --- | --- | --- | --- | --- | --- | --- |
| **№ of studies** | | **Study design** | | **Risk of bias** | | **Inconsistency** | | **Indirectness** | | **Imprecision** | | **Other considerations** | | | **nutritional screening** | | **no screening** |  |  |  |  |
| **Morbidity composite- anyone of cardiac, pulmonary, renal or surgical** | | | | | | | | | | | | | | | | | | | | | |
| 4  ([10-14](#_ENREF_10)) | observational studies | | serious | | not serious | | serious | | serious | | publication bias strongly suspected strong association all plausible residual confounding would reduce the demonstrated effect dose response gradient | | - | 748 | | 2040 | | | ⨁⨁⨁◯ MODERATE | | Important |
| **Reported Economic Financial Outcomes- including readmission rates** | | | | | | | | | | | | | | | | | | | | | |
| 4 ([15-18](#_ENREF_15)) | observational studies | | serious | | serious | | serious | | serious | | strong association all plausible residual confounding would suggest spurious effect, while no effect was observed | | 778 | | | 3056 | | | ⨁⨁◯◯ LOW | |  |

**5.Management of anemia**

**Question**: Should pre-operative anemia be managed in patients undergoing surgery of the spine

**Setting**: Perioperative patient care

| **Certainty assessment** | | | | | | | **№ of patients** | | **Certainty** | **Importance** | | |
| --- | --- | --- | --- | --- | --- | --- | --- | --- | --- | --- | --- | --- |
| **№ of studies** | **Study design** | **Risk of bias** | **Inconsistency** | **Indirectness** | **Imprecision** | **Other considerations** | **anaemia** | **no anaemia** |  |  |  |  |
| **Health service related outcome- Length of stay** | | | | | | | | | | | | |
| 2  ([19](#_ENREF_19), [20](#_ENREF_20)) | observational studies | very serious | serious | serious | serious | all plausible residual confounding would suggest spurious effect, while no effect was observed | 324 | 1223 | ⨁◯◯◯ VERY LOW | | Important |  |

**6. Peri-operative blood management**

**Question**: Intravenous tranexamic acid compared to placebo or no treatment for prevention/management of bleeding during surgery of the spine

**Setting**: Perioperative setting

| **Certainty assessment** | | | | | | | **№ of patients** | | **Certainty** | **Importance** |
| --- | --- | --- | --- | --- | --- | --- | --- | --- | --- | --- |
| **№ of studies** | **Study design** | **Risk of bias** | **Inconsistency** | **Indirectness** | **Imprecision** | **Other considerations** | **intravenous tranexamic acid** | **placebo or no treatment** |  |  |
|  | | | | | | | | | | |
| **Total Intraoperative blood loss** | | | | | | | | | | |
| 18  ([21-37](#_ENREF_21)) | observational studies | very serious | not serious | serious | very serious | strong association all plausible residual confounding would suggest spurious effect, while no effect was observed dose response gradient | 692 | 693 | ⨁⨁◯◯ LOW | Important |

| **Total perioperative blood transfusion** | | | | | | | | | | | |
| --- | --- | --- | --- | --- | --- | --- | --- | --- | --- | --- | --- |
| 18  -equivalent studies as above; | observational studies | very serious | not serious | serious | very serious | strong association all plausible residual confounding would suggest spurious effect, while no effect was observed dose response gradient | 692 | 693 | ⨁⨁◯◯ LOW | Important |  |

**6. Perioperative blood management**

**Question**: Cell saver use compared to routine care for patients undergoing moderate/ major spine surgery

**Setting**: Peri-operative patient care

| **Certainty assessment** | | | | | | | **№ of patients** | | **Certainty** | **Importance** | |
| --- | --- | --- | --- | --- | --- | --- | --- | --- | --- | --- | --- |
| **№ of studies** | **Study design** | **Risk of bias** | **Inconsistency** | **Indirectness** | **Imprecision** | **Other considerations** | **cell saver** | **routine care** |  |  |  |
| **Perioperative blood transfusion** | | | | | | | | | | | |
| 3  ([12](#_ENREF_12), [28](#_ENREF_28), [38](#_ENREF_38), [39](#_ENREF_39)) | observational studies | very serious | serious | serious | serious | all plausible residual confounding would suggest spurious effect, while no effect was observed | 68 | 72 | ⨁◯◯◯ VERY LOW | Important |  |

**12. Anaesthetic protocol**

**Question**: Total intravenous anaesthesia compared to volatile inhalation anaesthesia as a maintenance technique in surgery of the spine

**Setting**: Perioperative patient management

| **Certainty assessment** | | | | | | | **№ of patients** | | **Certainty** | **Importance** |
| --- | --- | --- | --- | --- | --- | --- | --- | --- | --- | --- |
| **№ of studies** | **Study design** | **Risk of bias** | **Inconsistency** | **Indirectness** | **Imprecision** | **Other considerations** | **total intravenous anaesthesia** | **volatile inhalation anaesthesia** |  |  |
| **VAS scores 24 hours post surgery;** | | | | | | | | | | |
| 2([40](#_ENREF_40), [41](#_ENREF_41)) | randomised trials | serious | not serious | not serious | serious | all plausible residual confounding would suggest spurious effect, while no effect was observed | 75 | 75 | ⨁⨁⨁◯ MODERATE | Important |

**16. Peri-operative analgesia**

**Question**: Continuous remifentanil infusion compared to standard therapy or placebo for patients undergoing surgery of the spine

**Setting**: Perioperative surgical setting

| **Certainty assessment** | | | | | | | **№ of patients** | | **Certainty** | **Importance** |
| --- | --- | --- | --- | --- | --- | --- | --- | --- | --- | --- |
| **№ of studies** | **Study design** | **Risk of bias** | **Inconsistency** | **Indirectness** | **Imprecision** | **Other considerations** | **continuous remifentanil infusion** | **standard therapy (no remi infusion0** |  |  |
| **Perioperative analgesic consumption within 24 hours of surgery** | | | | | | | | | | |
| 2 ([42](#_ENREF_42), [43](#_ENREF_43)) | randomized trials | very serious | serious | serious | serious | publication bias strongly suspected all plausible residual confounding would reduce the demonstrated effect dose response gradient | 55 | 35 | ⨁◯◯◯ VERY LOW | Important |

**16. Perioperative multimodal analgesia**

**Question**: Multimodal analgesia compared to standard care for ERSS?

**Setting**: perioperative patient management

| **Certainty assessment** | | | | | | | | | **№ of patients** | | **Certainty** | **Importance** |  |
| --- | --- | --- | --- | --- | --- | --- | --- | --- | --- | --- | --- | --- | --- |
| **№ of studies** | **Study design** | **Risk of bias** | **Inconsistency** | **Indirectness** | **Imprecision** | | | **Other considerations** | **multimodal analgesia** | **standard care** |  |  | |
| **Postoperative opioid consumption day 0-2;** | | | | | | | | | | | |  |  |
| 4  ([44-47](#_ENREF_44)) | observational studies | extremely serious | not serious | serious | serious | | strong association all plausible residual confounding would suggest spurious effect, while no effect was observed | | 235 | 428 | ⨁◯◯◯ VERY LOW | Important | |
| **Length of stay** | | | | | | | | | | | |  |  |
| 2  ([46](#_ENREF_46), [47](#_ENREF_47)) | observational studies | extremely serious | serious | serious | serious | strong association all plausible residual confounding would suggest spurious effect, while no effect was observed | | | 94 | 284 | ⨁◯◯◯ VERY LOW | Important | |
| **Postoperative Visual Analogue Scores(VAS) 24 hours;** | | | | | | | | | | | |  |  |
| 3 ([48](#_ENREF_48))  ([49](#_ENREF_49), [50](#_ENREF_50)) | randomized trials | serious | serious | not serious | not serious | all plausible residual confounding would reduce the demonstrated effect | | | 202 | 200 | ⨁⨁⨁◯ MODERATE | Important | |

**16. Question**: Should intravenous lignocaine infusion be used in patients undergoing surgery of the spine

**Setting**: Perioperative patient setting

| **Certainty assessment** | | | | | | | **№ of patients** | | **Certainty** | **Importance** |
| --- | --- | --- | --- | --- | --- | --- | --- | --- | --- | --- |
| **№ of studies** | **Study design** | **Risk of bias** | **Inconsistency** | **Indirectness** | **Imprecision** | **Other considerations** | **(**[**4**](#_ENREF_4)**)** | **(**[**13**](#_ENREF_13)**)** |  |  |
| **Pain management outcomes- Visual Analogue Scale (VAS) scores at 24 hours** | | | | | | | | | | |
| 5 ([51-54](#_ENREF_51))  ([50](#_ENREF_50), [55](#_ENREF_55)) | randomised trials | not serious | not serious | serious | serious | all plausible residual confounding would suggest spurious effect, while no effect was observed | 310 | 305 | ⨁⨁⨁◯ MODERATE | Important |

**21.Question**: What is the utility of evidence for early mobilization compared to standard care for patients undergoing surgery of the spine

**Setting**: Perioperative patient care

| **Certainty assessment** | | | | | | | **№ of patients** | | **Certainty** | | **Importance** | |
| --- | --- | --- | --- | --- | --- | --- | --- | --- | --- | --- | --- | --- |
| **№ of studies** | **Study design** | **Risk of bias** | **Inconsistency** | **Indirectness** | **Imprecision** | **Other considerations** | **early mobilization** | **standard care** |  |  |  |  |
| **Hospital related outcome- Length of stay** | | | | | | | | | | | | |
| 5  ([56-61](#_ENREF_56)) | observational studies | serious | not serious | serious | serious | publication bias strongly suspected strong association all plausible residual confounding would suggest spurious effect, while no effect was observed | 844 | 1864 | ⨁⨁⨁◯ MODERATE | Important | |  |

References:

1. Shea BJ, Reeves BC, Wells G, Thuku M, Hamel C, Moran J, et al. AMSTAR 2: a critical appraisal tool for systematic reviews that include randomised or non-randomised studies of healthcare interventions, or both. Bmj. 2017;358:j4008.

2. Glassman SD, Anagnost SC, Parker A, Burke D, Johnson JR, Dimar JR. The effect of cigarette smoking and smoking cessation on spinal fusion. Spine (Phila Pa 1976). 2000;25(20):2608-15.

3. Andersen T, Christensen FB, Laursen M, Hoy K, Hansen ES, Bunger C. Smoking as a predictor of negative outcome in lumbar spinal fusion. Spine (Phila Pa 1976). 2001;26(23):2623-8.

4. Bydon M, De la Garza-Ramos R, Abt NB, Gokaslan ZL, Wolinsky JP, Sciubba DM, et al. Impact of smoking on complication and pseudarthrosis rates after single- and 2-level posterolateral fusion of the lumbar spine. Spine (Phila Pa 1976). 2014;39(21):1765-70.

5. Lotzke H, Brisby H, Gutke A, Hagg O, Jakobsson M, Smeets R, et al. A Person-Centered Prehabilitation Program Based on Cognitive-Behavioral Physical Therapy for Patients Scheduled for Lumbar Fusion Surgery - A Randomized Controlled Trial. Phys Ther. 2019.

6. Louw A, Diener I, Landers MR, Puentedura EJ. Preoperative pain neuroscience education for lumbar radiculopathy: a multicenter randomized controlled trial with 1-year follow-up. Spine (Phila Pa 1976). 2014;39(18):1449-57.

7. Lindback Y, Tropp H, Enthoven P, Abbott A, Oberg B. PREPARE: presurgery physiotherapy for patients with degenerative lumbar spine disorder: a randomized controlled trial. Spine J. 2018;18(8):1347-55.

8. Nielsen PR, Jorgensen LD, Dahl B, Pedersen T, Tonnesen H. Prehabilitation and early rehabilitation after spinal surgery: randomized clinical trial. Clin Rehabil. 2010;24(2):137-48.

9. Rolving N, Nielsen CV, Christensen FB, Holm R, Bunger C, Ostergaard L. Does a preoperative cognitive-behavioural intervention affect postsurgical pain, mobilisation and length of hospitalisation in lumbar spinal fusion patients? European spine journal. 2014;23:S572.

10. Adogwa O, Carr K, Thompson P, Hoang K, Darlington T, Perez E, et al. A prospective, multi-institutional comparative effectiveness study of lumbar spine surgery in morbidly obese patients: does minimally invasive transforaminal lumbar interbody fusion result in superior outcomes? World Neurosurg. 2015;83(5):860-6.

11. Adogwa O, Elsamadicy AA, Sergesketter A, Vuong VD, Mehta AI, Vasquez RA, et al. Prophylactic use of intraoperative vancomycin powder and postoperative infection: an analysis of microbiological patterns in 1200 consecutive surgical cases. J Neurosurg Spine. 2017;27(3):328-34.

12. Development of an Enhanced Recovery After Surgery (ERAS) approach for lumbar spinal fusion. J Neurosurg Spine. 2017;26(4):411-8.

13. Fu MC, Buerba RA, Grauer JN. Preoperative Nutritional Status as an Adjunct Predictor of Major Postoperative Complications Following Anterior Cervical Discectomy and Fusion. Clin Spine Surg. 2016;29(4):167-72.

14. Guan J, Cole CD, Schmidt MH, Dailey AT. Utility of intraoperative rotational thromboelastometry in thoracolumbar deformity surgery. J Neurosurg Spine. 2017;27(5):528-33.

15. Jevsevar DS, Karlin LI. The relationship between preoperative nutritional status and complications after an operation for scoliosis in patients who have cerebral palsy. J Bone Joint Surg Am. 1993;75(6):880-4.

16. Phan K, Kim JS, Xu J, Di Capua J, Lee NJ, Kothari P, et al. Nutritional Insufficiency as a Predictor for Adverse Outcomes in Adult Spinal Deformity Surgery. Global Spine J. 2018;8(2):164-71.

17. Salvetti DJ, Tempel ZJ, Gandhoke GS, Parry PV, Grandhi RM, Kanter AS, et al. Preoperative prealbumin level as a risk factor for surgical site infection following elective spine surgery. Surg Neurol Int. 2015;6(Suppl 19):S500-3.

18. Salvetti DJ, Tempel ZJ, Goldschmidt E, Colwell NA, Angriman F, Panczykowski DM, et al. Low preoperative serum prealbumin levels and the postoperative surgical site infection risk in elective spine surgery: a consecutive series. J Neurosurg Spine. 2018;29(5):549-52.

19. Khanna R, Harris DA, McDevitt JL, Fessler RG, Carabini LM, Lam SK, et al. Impact of Anemia and Transfusion on Readmission and Length of Stay After Spinal Surgery: A Single-center Study of 1187 Operations. Clin Spine Surg. 2017;30(10):E1338-e42.

20. Sanoufa M, Smisson W, Floyd H, Robinson JS. The effect of anaemia on hospital length of stay in lumbar decompression and fusion procedures. J Perioper Pract. 2015;25(12):267-71.

21. Choi HY, Hyun S-J, Kim K-J, Jahng T-A, Kim H-J. Effectiveness and Safety of Tranexamic Acid in Spinal Deformity Surgery. J Korean Neurosurg Soc. 2017;60(1):75-81.

22. Kushioka J, Yamashita T, Okuda S, Maeno T, Matsumoto T, Yamasaki R, et al. High-dose tranexamic acid reduces intraoperative and postoperative blood loss in posterior lumbar interbody fusion. J Neurosurg Spine. 2017;26(3):363-7.

23. Yu C-C, Gao W-J, Yang J-S, Gu H, Zhu M, Sun K, et al. Can tranexamic acid reduce blood loss in cervical laminectomy with lateral mass screw fixation and bone grafting: a retrospective observational study. Medicine (Baltimore). 2017;96(5):e6043-e.

24. Sui W-y, Ye F, Yang J-l. Efficacy of tranexamic acid in reducing allogeneic blood products in adolescent idiopathic scoliosis surgery. BMC Musculoskeletal Disorders. 2016;17(1):187.

25. Ng BK, Chau WW, Hung AL, Hui AC, Lam TP, Cheng JC. Use of Tranexamic Acid (TXA) on reducing blood loss during scoliosis surgery in Chinese adolescents. Scoliosis. 2015;10:28.

26. Xie J, Lenke LG, Li T, Si Y, Zhao Z, Wang Y, et al. Preliminary investigation of high-dose tranexamic acid for controlling intraoperative blood loss in patients undergoing spine correction surgery. Spine J. 2015;15(4):647-54.

27. da Rocha VM, de Barros AG, Naves CD, Gomes NL, Lobo JC, Villela Schettino LC, et al. Use of tranexamic acid for controlling bleeding in thoracolumbar scoliosis surgery with posterior instrumentation. Rev Bras Ortop. 2015;50(2):226-31.

28. Naik BI, Pajewski TN, Bogdonoff DI, Zuo Z, Clark P, Terkawi AS, et al. Rotational thromboelastometry-guided blood product management in major spine surgery. J Neurosurg Spine. 2015;23(2):239-49.

29. Khurana G, Jindal P, Sharma JP, Bansal KK. Postoperative pain and long-term functional outcome after administration of gabapentin and pregabalin in patients undergoing spinal surgery. Spine (Phila Pa 1976). 2014;39(6):E363-8.

30. Dhawale AA, Shah SA, Sponseller PD, Bastrom T, Neiss G, Yorgova P, et al. Are antifibrinolytics helpful in decreasing blood loss and transfusions during spinal fusion surgery in children with cerebral palsy scoliosis? Spine (Phila Pa 1976). 2012;37(9):E549-55.

31. Shapiro F, Zurakowski D, Sethna NF. Tranexamic acid diminishes intraoperative blood loss and transfusion in spinal fusions for duchenne muscular dystrophy scoliosis. Spine (Phila Pa 1976). 2007;32(20):2278-83.

32. Bednar DA, Bednar VA, Chaudhary A, Farrokhyar F. Tranexamic acid for hemostasis in the surgical treatment of metastatic tumors of the spine. Spine (Phila Pa 1976). 2006;31(8):954-7.

33. Baldus CR, Bridwell KH, Lenke LG, Okubadejo GO. Can we safely reduce blood loss during lumbar pedicle subtraction osteotomy procedures using tranexamic acid or aprotinin? A comparative study with controls. Spine (Phila Pa 1976). 2010;35(2):235-9.

34. Endres S, Heinz M, Wilke A. Efficacy of tranexamic acid in reducing blood loss in posterior lumbar spine surgery for degenerative spinal stenosis with instability: a retrospective case control study. BMC Surg. 2011;11:29.

35. Newton PO, Bastrom TP, Emans JB, Shah SA, Shufflebarger HL, Sponseller PD, et al. Antifibrinolytic agents reduce blood loss during pediatric vertebral column resection procedures. Spine (Phila Pa 1976). 2012;37(23):E1459-63.

36. Lykissas MG, Crawford AH, Chan G, Aronson LA, Al-Sayyad MJ. The effect of tranexamic acid in blood loss and transfusion volume in adolescent idiopathic scoliosis surgery: a single-surgeon experience. J Child Orthop. 2013;7(3):245-9.

37. Yagi M, Hasegawa J, Nagoshi N, Iizuka S, Kaneko S, Fukuda K, et al. Does the intraoperative tranexamic acid decrease operative blood loss during posterior spinal fusion for treatment of adolescent idiopathic scoliosis? Spine (Phila Pa 1976). 2012;37(21):E1336-42.

38. Buell TJ, Taylor DG, Chen CJ, Dunn LK, Mullin JP, Mazur MD, et al. Rotational thromboelastometry-guided transfusion during lumbar pedicle subtraction osteotomy for adult spinal deformity: preliminary findings from a matched cohort study. Neurosurg Focus. 2019;46(4):E17.

39. Guan J, Holland CM, Schmidt MH, Dailey AT, Mahan MA, Bisson EF. Association of low perioperative prealbumin level and surgical complications in long-segment spinal fusion patients: A retrospective cohort study. Int J Surg. 2017;39:135-40.

40. Konstantopoulos K, Makris A, Moustaka A, Karmaniolou I, Konstantopoulos G, Mela A. Sevoflurane versus propofol anesthesia in patients undergoing lumbar spondylodesis: a randomized trial. J Surg Res. 2013;179(1):72-7.

41. Lin WL, Lee MS, Wong CS, Chan SM, Lai HC, Wu ZF, et al. Effects of intraoperative propofol-based total intravenous anesthesia on postoperative pain in spine surgery: Comparison with desflurane anesthesia - a randomised trial. Medicine (Baltimore). 2019;98(13):e15074.

42. Yeom JH, Kim KH, Chon MS, Byun J, Cho SY. Remifentanil used as adjuvant in general anesthesia for spinal fusion does not exhibit acute opioid tolerance. Korean J Anesthesiol. 2012;63(2):103-7.

43. Crawford MW, Hickey C, Zaarour C, Howard A, Naser B. Development of acute opioid tolerance during infusion of remifentanil for pediatric scoliosis surgery. Anesth Analg. 2006;102(6):1662-7.

44. Rajpal S, Gordon DB, Pellino TA, Strayer AL, Brost D, Trost GR, et al. Comparison of perioperative oral multimodal analgesia versus IV PCA for spine surgery. J Spinal Disord Tech. 2010;23(2):139-45.

45. Mathiesen O, Dahl B, Thomsen BA, Kitter B, Sonne N, Dahl JB, et al. A comprehensive multimodal pain treatment reduces opioid consumption after multilevel spine surgery. Eur Spine J. 2013;22(9):2089-96.

46. Singh K, Bohl DD, Ahn J, Massel DH, Mayo BC, Narain AS, et al. Multimodal Analgesia Versus Intravenous Patient-Controlled Analgesia for Minimally Invasive Transforaminal Lumbar Interbody Fusion Procedures. Spine (Phila Pa 1976). 2017;42(15):1145-50.

47. Bohl DD, Louie PK, Shah N, Mayo BC, Ahn J, Kim TD, et al. Multimodal Versus Patient-Controlled Analgesia After an Anterior Cervical Decompression and Fusion. Spine (Phila Pa 1976). 2016;41(12):994-8.

48. Garcia RM, Cassinelli EH, Messerschmitt PJ, Furey CG, Bohlman HH. A multimodal approach for postoperative pain management after lumbar decompression surgery: a prospective, randomized study. J Spinal Disord Tech. 2013;26(6):291-7.

49. Kim SI, Ha KY, Oh IS. Preemptive multimodal analgesia for postoperative pain management after lumbar fusion surgery: a randomized controlled trial. Eur Spine J. 2016;25(5):1614-9.

50. Maheshwari K, Avitsian R, Sessler DI, Makarova N, Tanios M, Raza S, et al. Multimodal Analgesic Regimen for Spine Surgery: A Randomized Placebo-controlled Trial. Anesthesiology. 2020;132(5):992-1002.

51. Batko I, Kościelniak-Merak B, Tomasik PJ, Kobylarz K, Wordliczek J. Lidocaine as an element of multimodal analgesic therapy in major spine surgical procedures in children: a prospective, randomized, double-blind study. Pharmacol Rep. 2020.

52. Kim KT, Cho DC, Sung JK, Kim YB, Kang H, Song KS, et al. Intraoperative systemic infusion of lidocaine reduces postoperative pain after lumbar surgery: a double-blinded, randomized, placebo-controlled clinical trial. Spine J. 2014;14(8):1559-66.

53. Farag E, Ghobrial M, Sessler DI, Dalton JE, Liu J, Lee JH, et al. Effect of perioperative intravenous lidocaine administration on pain, opioid consumption, and quality of life after complex spine surgery. Anesthesiology. 2013;119(4):932-40.

54. Ibrahim A, Aly M, Farrag W. Effect of intravenous lidocaine infusion on long-term postoperative pain after spinal fusion surgery. Medicine (Baltimore). 2018;97(13):e0229.

55. Dewinter G, Moens P, Fieuws S, Vanaudenaerde B, Van de Velde M, Rex S. Systemic lidocaine fails to improve postoperative morphine consumption, postoperative recovery and quality of life in patients undergoing posterior spinal arthrodesis. A double-blind, randomized, placebo-controlled trial. Br J Anaesth. 2017;118(4):576-85.

56. Soffin EM, Vaishnav AS, Wetmore D, Barber L, Hill P, Gang CH, et al. Design and Implementation of an Enhanced Recovery After Surgery (ERAS) Program for Minimally Invasive Lumbar Decompression Spine Surgery: Initial Experience. Spine (Phila Pa 1976). 2018.

57. Soffin EM, Wetmore DS, Barber LA, Vaishnav AS, Beckman JD, Albert TJ, et al. An enhanced recovery after surgery pathway: association with rapid discharge and minimal complications after anterior cervical spine surgery. Neurosurg Focus. 2019;46(4):E9.

58. Gornitzky AL, Flynn JM, Muhly WT, Sankar WN. A Rapid Recovery Pathway for Adolescent Idiopathic Scoliosis That Improves Pain Control and Reduces Time to Inpatient Recovery After Posterior Spinal Fusion. Spine Deform. 2016;4(4):288-95.

59. Muhly WT, Sankar WN, Ryan K, Norton A, Maxwell LG, DiMaggio T, et al. Rapid Recovery Pathway After Spinal Fusion for Idiopathic Scoliosis. Pediatrics. 2016;137(4).

60. Sivaganesan A, Wick JB, Chotai S, Cherkesky C, Stephens BF, Devin CJ. Perioperative Protocol for Elective Spine Surgery Is Associated With Reduced Length of Stay and Complications. J Am Acad Orthop Surg. 2019;27(5):183-9.

61. Bradywood A, Farrokhi F, Williams B, Kowalczyk M, Blackmore CC. Reduction of Inpatient Hospital Length of Stay in Lumbar Fusion Patients With Implementation of an Evidence-Based Clinical Care Pathway. Spine (Phila Pa 1976). 2017;42(3):169-76.
