# Supplementary material for: Pathway for enhanced recovery after spinal surgery-a systematic review of evidence for use of individual components
Source: BMC Anesthesiol. 2021 Mar 10;21:74. doi: 10.1186/s12871-021-01281-1 (PMC7944908; doi:10.1186/s12871-021-01281-1)
Supplement: Supplementary file 4 — Additional file 4: Summary of evidence for each component. [file 12871_2021_1281_MOESM4_ESM.docx]

*Summary of evidence level for each of the 22 components of ERSS*

| *ERAS Element* | *Evidence level* |
| --- | --- |
| 1.Preadmission information, education and counseling | There is limited specific evidence for the intervention in surgery of the spine; |
| 2.Risk assessment, preoperative optimization, including lifestyle factor modification |  |
| 2.1 Preoperative risk stratification | Moderate quality evidence in support of individualized risk stratification tool; |
| 2.2 Preoperative assessment and optimization | Low quality due to a limited number of heterogenous studies; |
| 2.3 Alcohol use | Moderate quality due to heterogenous endpoints; |
| 2.4 Tobacco use | High quality evidence base; |
| 3.Prehabilitation | Low quality evidence for impact of multimodal prehabilitation; |
| 4. Preoperative Nutritional Care |  |
| 4.1. Nutritional Assessment and Screening | Moderate quality for risk assessment; |
| 4.2 Immune-nutrition | Very low quality evidence; |
| 5. Management of anaemia | Moderate quality for correcting iron deficiency anaemia; |
| 6. Perioperative blood conservation strategies | High quality for antifibrinolytic use when indicated;  Moderate quality for cell saver use when significant blood loss is anticipated;  Low quality for use of point of care testing; |
| 7.Preoperative fasting and carbohydrate loading | High quality for adherence to standardized societal fasting guidelines; |
| 8. Pre-emptive analgesia | High quality for pre-emptive gabapentinoid administration;  Moderate quality for pre-emptive multimodal regimes;  Low quality for individual pre-emptive NSAID administration; |
| 9. Prevention of postoperative nausea and vomiting | High quality for risk stratification;  High quality for anaesthetic techniques and antiemetic use as per recommended societal guidelines;  Moderate quality for anaesthetic techniques which minimize risk of PONV |
| 10. Surgical site preparation and prophylaxis |  |
| 10.1 Surgical site preparation | High quality for using the alcohol-based skin preparations;  Moderate quality for decreasing the bacterial load with CHG; |
| 10.2 Antimicrobial prophylaxis | High quality for intra-operative antibiotic prophylaxis;  Moderate quality evidence for use of intravenous vancomycin in patients at risk of MRSA;  Moderate quality for use of intraoperative vancomycin powder; |
| 11.Local anaesthetic infiltration | Moderate quality; |
| 12. Standard Anaesthetic protocol | Moderate quality for total intravenous anaesthesia use in spine surgery;  Low quality for continuous intra-operative remifentanil infusion use in spine surgery; |
| 13. Surgical access- open and minimally invasive spinal surgery | Moderate quality for the intervention when clinically appropriate; |
| 14. Maintenance of normothermia | Moderate quality for maintenance of intraoperative normothermia; |
| 15. Intraoperative fluid and electrolyte therapy | Low quality for goal-directed intraoperative fluid management; |
| 16. Peri-operative analgesia | Moderate quality for acetaminophen and NSAID administration; Moderate quality for intraoperative and postoperative ketamine administration;  Moderate quality for intraoperative intrathecal morphine administration;  Very low quality for perioperative methadone administration;  Very low quality alpha-2 receptor agonists;  Moderate quality for intravenous intraoperative lignocaine infusion;  Moderate quality for multimodal analgesic regimens; |
| 17. Thromboprophylaxis | Moderate quality for postoperative mechanical thromboprophylaxis;  Low quality for postoperative chemical thromboprophylaxis; |
| 18. Urinary drainage | Moderate quality for urinary catheter removal within 48 hours after surgery; |
| 19. Post-operative nutrition and fluid management | Moderate quality for early transition to oral intake;  Low quality for goal-directed fluid management;  Low quality for goal-orientated post-operative fluid management; |
| 20. Post-operative glycemic control | Low quality for conventional blood glucose control; |
| 21. Early mobilization | Low quality due to observational structure of available studies; |
| 22.Audit | Low quality; |
