# Supplementary material for: Pathway for enhanced recovery after spinal surgery-a systematic review of evidence for use of individual components
Source: BMC Anesthesiol. 2021 Mar 10;21:74. doi: 10.1186/s12871-021-01281-1 (PMC7944908; doi:10.1186/s12871-021-01281-1)
Supplement: Supplementary file 5 — Additional file 5: Forest plots. [file 12871_2021_1281_MOESM5_ESM.docx]

**Forest Plots**

Forrest plots were generated for the following pathway components which allowed for pooling of heterogenous results in randomized controlled trials. Please note there was either significant clinical or statistical clinical heterogeneity. Results of the meta-analysis of each component were used to inform the thematic synthesis of data.

12. Anaesthetic technique

Question: Total intravenous anaesthesia compared to volatile inhalation anaesthesia as a maintenance technique in surgery of the spine

Setting: Perioperative patient management

Question: Continuous remifentanil infusion compared to standard therapy or placebo for patients undergoing surgery of the spine

Setting: Perioperative surgical setting


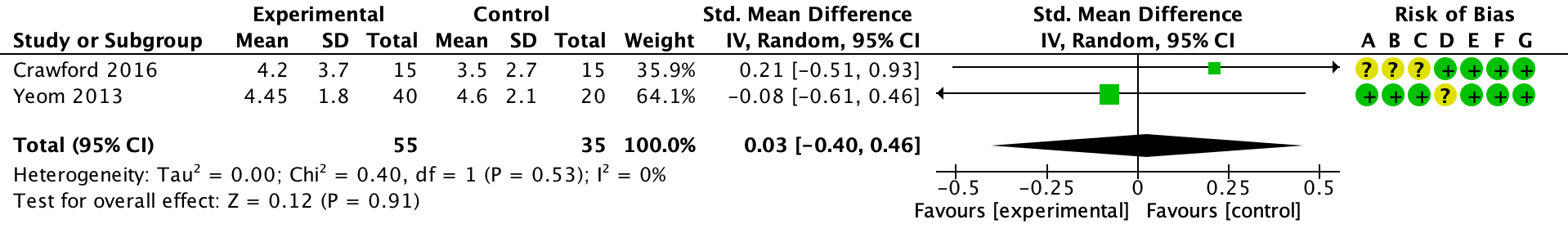


16. Question: Multimodal analgesia efficacy in lowering pain scores compared to standard care for ERSS?

Setting: perioperative patient management

Outcome: VAS at 24 hours


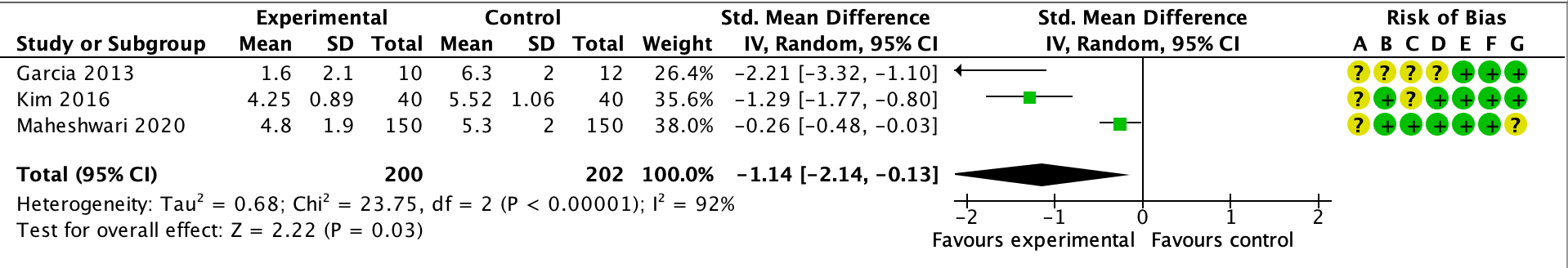


16. Question: Should intravenous lignocaine infusion be used in patients undergoing surgery of the spine

Setting: Perioperative patient setting

Outcome: VAS at 24 hours


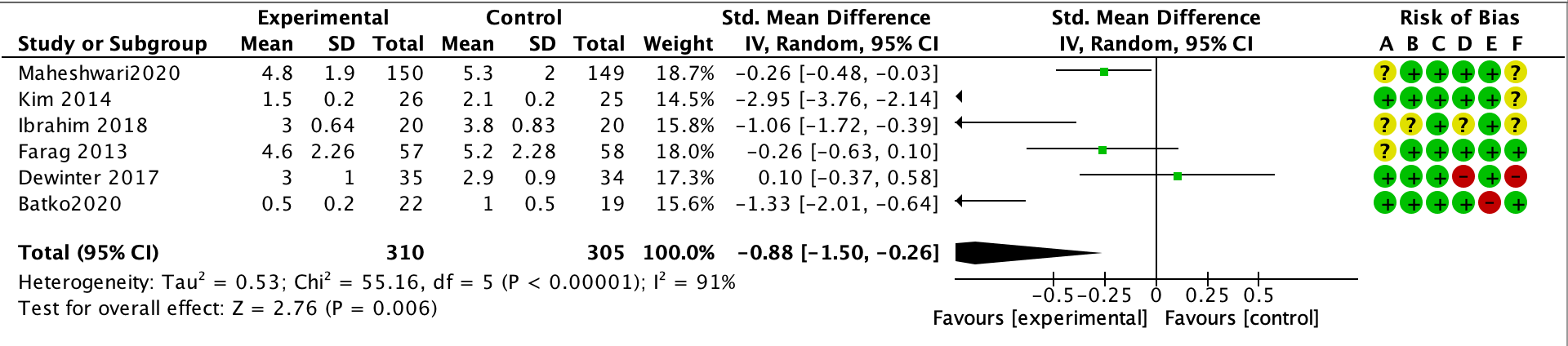


Forest plots were not generated for Evidence Profile Tables outlining randomized controlled trials with heterogenous endpoints/duration of follow up

**3. Prehabilitation**

Question: Prehabilitation compared to standard care for patients undergoing and enhanced recovery pathway in surgery of the spine;

**Forrest Plots were not generated for observational studies summarized in Evidence Profile Tables.**

2.4 Cessation of tobacco use

Question: Do smokers have a higher risk of non-union/surgical complications after surgery of the spine?

3. Prehabilitation

Question: Does prehabilitation decrease the length of stay?

4.1. Nutritional screening

Question: Nutritional screening compared to no screening in patients undergoing spinal surgery?

5. Peri-operative anaemia management

Question: Should pre-operative anaemia be managed in patients undergoing surgery of the spine?

6. Peri-operative blood management, observational component

Question: Does tranexamic acid use decrease total peri-operative blood loss?

Question: Does tranexamic acid use decrease total blood transfusion?

6. Peri-operative blood management, observational component

Question: Cell saver use compared to routine care for patients undergoing moderate/ major spine surgery

10.2 Question: Vancomycin powder intra-wound administration compared to standard therapy or placebo for spinal surgery

13. Question: Minimally invasive surgery compared to standard care for enhanced recovery pathways in surgery of the spine

Setting: Perioperative patient management

16. Question: Multimodal analgesia compared to standard care for ERSS?

Setting: perioperative patient management

21.Question: What is the utility of evidence for early mobilization compared to standard care for patients undergoing surgery of the spine

Setting: Perioperative patient care

|  |
| --- |

1. Glassman SD, Anagnost SC, Parker A, Burke D, Johnson JR, Dimar JR. The effect of cigarette smoking and smoking cessation on spinal fusion. Spine (Phila Pa 1976). 2000;25(20):2608-15.

2. Andersen T, Christensen FB, Laursen M, Hoy K, Hansen ES, Bunger C. Smoking as a predictor of negative outcome in lumbar spinal fusion. Spine (Phila Pa 1976). 2001;26(23):2623-8.

3. Bydon M, De la Garza-Ramos R, Abt NB, Gokaslan ZL, Wolinsky JP, Sciubba DM, et al. Impact of smoking on complication and pseudarthrosis rates after single- and 2-level posterolateral fusion of the lumbar spine. Spine (Phila Pa 1976). 2014;39(21):1765-70.

4. Lotzke H, Brisby H, Gutke A, Hagg O, Jakobsson M, Smeets R, et al. A Person-Centered Prehabilitation Program Based on Cognitive-Behavioral Physical Therapy for Patients Scheduled for Lumbar Fusion Surgery - A Randomized Controlled Trial. Phys Ther. 2019.

5. Louw A, Diener I, Landers MR, Puentedura EJ. Preoperative pain neuroscience education for lumbar radiculopathy: a multicenter randomized controlled trial with 1-year follow-up. Spine (Phila Pa 1976). 2014;39(18):1449-57.

6. Lindback Y, Tropp H, Enthoven P, Abbott A, Oberg B. PREPARE: presurgery physiotherapy for patients with degenerative lumbar spine disorder: a randomized controlled trial. Spine J. 2018;18(8):1347-55.

7. Nielsen PR, Jorgensen LD, Dahl B, Pedersen T, Tonnesen H. Prehabilitation and early rehabilitation after spinal surgery: randomized clinical trial. Clin Rehabil. 2010;24(2):137-48.

8. Rolving N, Nielsen CV, Christensen FB, Holm R, Bunger C, Ostergaard L. Does a preoperative cognitive-behavioural intervention affect postsurgical pain, mobilisation and length of hospitalisation in lumbar spinal fusion patients? European spine journal. 2014;23:S572.

9. Batko I, Kościelniak-Merak B, Tomasik PJ, Kobylarz K, Wordliczek J. Lidocaine as an element of multimodal analgesic therapy in major spine surgical procedures in children: a prospective, randomized, double-blind study. Pharmacol Rep. 2020.

10. Kim KT, Cho DC, Sung JK, Kim YB, Kang H, Song KS, et al. Intraoperative systemic infusion of lidocaine reduces postoperative pain after lumbar surgery: a double-blinded, randomized, placebo-controlled clinical trial. Spine J. 2014;14(8):1559-66.

11. Farag E, Ghobrial M, Sessler DI, Dalton JE, Liu J, Lee JH, et al. Effect of perioperative intravenous lidocaine administration on pain, opioid consumption, and quality of life after complex spine surgery. Anesthesiology. 2013;119(4):932-40.

12. Ibrahim A, Aly M, Farrag W. Effect of intravenous lidocaine infusion on long-term postoperative pain after spinal fusion surgery. Medicine (Baltimore). 2018;97(13):e0229.

13. Dewinter G, Moens P, Fieuws S, Vanaudenaerde B, Van de Velde M, Rex S. Systemic lidocaine fails to improve postoperative morphine consumption, postoperative recovery and quality of life in patients undergoing posterior spinal arthrodesis. A double-blind, randomized, placebo-controlled trial. Br J Anaesth. 2017;118(4):576-85.

14. Maheshwari K, Avitsian R, Sessler DI, Makarova N, Tanios M, Raza S, et al. Multimodal Analgesic Regimen for Spine Surgery: A Randomized Placebo-controlled Trial. Anesthesiology. 2020;132(5):992-1002.
